# Carotid Artery Occlusion by Rhinoorbitocerebral Mucormycosis

**DOI:** 10.1155/2012/812420

**Published:** 2012-10-30

**Authors:** Faisal Al-Otaibi, Monirah Albloushi, Hindi Alhindi, Michael S. Timms

**Affiliations:** ^1^Division of Neurosurgery, Neurosciences Department, King Faisal Specialist Hospital and Research Center, Riyadh 11211, Saudi Arabia; ^2^College of Nursing, King Saud University, Riyadh 11211, Saudi Arabia; ^3^Department of Pathology and Laboratory Medicine, King Faisal Specialist Hospital and Research Center, Riyadh 11211, Saudi Arabia; ^4^Department of Otolaryngology, King Faisal Specialist Hospital and Research Center, Riyadh 11211, Saudi Arabia

## Abstract

Mucormycosis is the third most common invasive fungal infection that particularly occurs in immunocompromised patients. Intracranial and extracranial arteriovenous vasculopathy is a complication that makes this disease more complex and difficult to treat. We describe a 23-year-old female, who presented to her local hospital with acute blindness and diabetic ketoacidosis-induced coma requiring intensive care treatment. She was found to have lesions in the nasal sinuses, orbit, and frontal base. The left carotid artery was occluded from its origin in the neck to the supraclinoid segment and left cavernous sinus involvement. No cerebral infarction was noted. Biopsies obtained by endonasal debridement confirmed mucormycosis. In addition to antimicrobial therapy, she underwent several multidisciplinary approaches to treat her disease. Multiple endonasal, and cranial procedures were done including bilateral orbital exenteration. After prolonged treatment on the intensive care unit she made a remarkable recovery to the point where she was communicating verbally and had normal limb movements and later discharged home. She remained alive and well for two months, but later succumbed to a recurrence of her disease. In conclusion, mucormycosis-induced vasculopathy is a complex problem, which merits aggressive treatment of this invasive disease. It is normally regarded as an indicator of grave prognosis.

## 1. Introduction

Mucormycosis is an acute, angioinvasive infection that usually occurs in immunocompromised patients [1]. The mortality rate is found to be more than 60% [1, 2]. There are different forms of mucormycosis which include pulmonary, cutaneous, gastrointestinal, rhinocerebral, and disseminated [2]. The ideal host of this aggressive fungal infection is a patient with diabetic ketoacidosis, renal failure associated uremia, chronic steroid therapy, and neutropenia [3]. Diagnosis is usually achieved from microbiology and histopathology studies. Clinical and radiological suspicion is the key for early diagnosis.

In rhinocerebral infection, the organism is inhaled into the paranasal sinuses and spreads locally to orbit and brain [3, 4]. Clinical features are fever, reduced sensorium, diplopia, and blindness. In cases with severe underlying disease and in immunocompromised patient, mortality can occur in few days [2]. The disease is known to be angioinvasive causing thrombosis within large venous channels and arteries. Vasculopathy of the intracranial arteries and veins can result in stroke [5].

Herein, we present an immunocompromised patient with rhinoorbitocerebral mucormycosis associated with asymptomatic complete occlusion of the extracranial and intracranial left carotid artery.

## 2. Case Report

We report a 23-year-old Arabic female, known to have diabetes mellitus type 1 on insulin. She presented to her local hospital with reduced level of consciousness, seizure, and bilateral visual loss. She was found to have diabetic ketoacidosis associated with acute renal failure and uremia. Cranial images depicted extensive lesions in the paranasal sinuses and frontal cranial base. Biopsies obtained by endonasal debridement confirmed the diagnosis of mucormycosis. Afterward, she was transferred to our institution and admitted to the intensive care unit ventilated and on renal dialysis. On examination, she was had high grade fever with stable hemodynamic status and on pressure support ventilation. Neurologically, she had reduced sensorium with Glasgow coma score of 10 over 15 points and bilateral dilated pupils with no reaction to light. There was mucoid from both eyes and nostrils. Laboratory investigations revealed leukocytosis with neutrophilia, uremia, elevated liver enzymes, and features of diabetic ketoacidosis. Septic screen revealed multidrug-resistant pseudomonas in the respiratory culture and the eye secretions. This was sensitive to colistin. She was kept on amphotericin B, vancomycin, colistin, and nystatin. 

**Figure 1:**
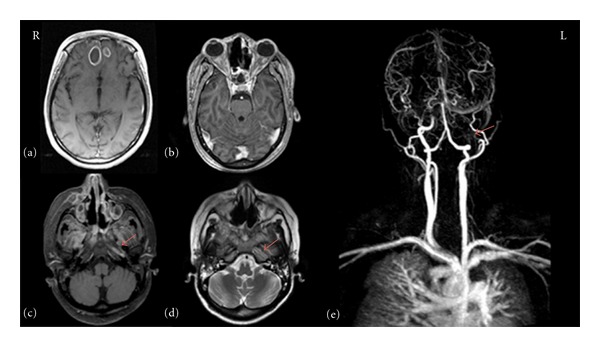
(a) MRI brain, axial T1 weighted image with gadolinium depicting anterior frontal bilateral abscesses. (b) MRI brain, axial T1 showing the orbital disease. (c) MRI brain, axial T1 demonstrating gadolinium enhancement around the left carotid artery canal. (d) T2 axial image demonstrating the absence of signal void at the left carotid artery. (e) MR angiography depicting complete occlusion of the left internal carotid artery cervical and cranial base segment.

Initially she was treated for the diabetic ketoacidosis, and the acute renal failure and these conditions were stabilized. Computerized tomography (CT) of paranasal sinuses and magnetic resonance image (MRI) of the brain revealed extensive lesions in the paranasal sinuses, both orbits, and mesial anterior frontal lobe (Figure 1). The left internal carotid artery showed complete occlusion from its cervical origin to the supraclinoid segment, and there was evidence of good collateral supply from the right carotid system through the circle of willis (Figure 1). The left cavernous sinus was also involved with the disease, and other cerebral venous systems were found to be patent.

**Figure 2:**
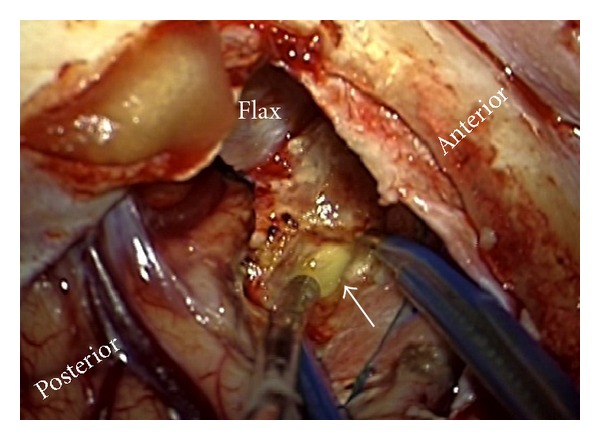
Intraoperative photo post right frontal craniotomy craniotomy demonstrating the abscess at the anterior frontal above the ethmoid sinus (arrow).

**Figure 3:**
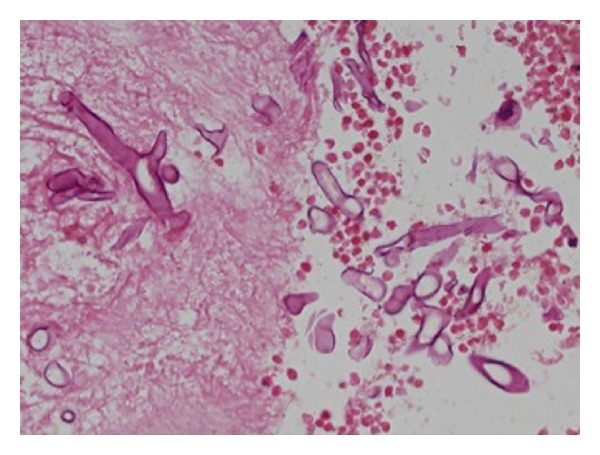
Histopathology slide demonstrating broad nonseptate hyphae in longitudinal (upper left) and cross-sectional (right) profiles. Eosin and Hematoxylin stain. Original magnification ×400.

She underwent further endonasal endoscopic exploration and debridement of the paranasal sinuses in two subsequent operative sessions. After this initial treatment, she improved in terms of consciousness and comprehension. She was found to have bilateral blindness, and she was moving all limbs with no lateralizing weakness. Follow-up MRI of the brain and orbits showed no improvement of the orbitocerebral disease plus cerebral abscess formation. In the context of bilateral blindness and extensive bilateral orbital and anterior cerebral disease, she underwent bifrontal craniotomy, evacuation of the cerebral abscesses in addition to bilateral orbital exenteration, and sinuses debridement (Figures 2 and 3).

Postoperatively, she was kept on antimicrobial agents and hyperbaric oxygen, of which she received forty sessions, twice per day over three weeks. Subsequently, she had a transient clinical and radiological improvement over two weeks followed by progression of the local sinuses and cerebral disease. 

The last brain MRI showed diffuse leptomeningeal with multiple parenchymal diseases. She was therefore transferred back to her local hospital for comfort care only. She then improved clinically and was able to go home, but suffered a relapse after two months and subsequently died.

## 3. Discussion

Rhino-orbito-cerebral mucormycosis (ROCM) is a rare aggressive fungal infection of the paranasal sinuses and brain. This infection is acquired by inhalation, ingestion, or it can be by a direct fungal contact to an open wounds [6]. Mucormycosis in general represents up to 13% of all fungal infections in hematology patients autopsy [7]. Usually mucormycosis affects immunocompromised patients such as in case of hematological malignancy, organ transplant, burns, diabetes mellitus, and chronic high-dose steroid therapy [3]. Interestingly, mucormycosis is not common in human immunodeficiency virus (HIV) infected patients, which might be due to the presence of neutrophils that carries the defense mechanism against mucormycosis rather than T lymphocytes [8]. It rarely affects immunocompetent patients [9]. Early diagnosis and treatment are critical to increase the chance of survival. In 2001, Chakrabarti and colleagues reported on 120 cases of mucormycosis infection [10]. They found that rhinocerebral and cutaneous infections are diagnosed ante mortem in 91% and 100% of cases, respectively, compared to 31% in pulmonary and 44% in renal involvement.

Rhino-orbito-cerebral infection starts usually in the paranasal sinuses and progress to involve the orbit and the brain [11]. Internal carotid artery and cavernous sinus thrombosis have been reported complication of this disease [2, 12, 13]. Thajeb and colleagues reported on 6 cases with fatal cerebrovascular accident related to cerebral arteries thrombosis among treated mucormycosis cases in three centers over a period between 1994 and 2003 [5]. Four patients developed internal carotid artery stroke and one with combined carotid and basilar and one with isolated basilar system stroke. In this report, one of the patients developed multiple cerebral infarctions associated with subarachnoid hemorrhage. In our patient, there is evidence of thrombosis involving the long segment of the left internal carotid artery. Despite internal carotid artery occlusion, there was no clinical or radiological signs of stroke. The presence of good collateral blood supply via the circle of willis appears to protect against the occurrence of cerebral ischemia. The involvement of the left cavernous sinus can cause ophthalmoplegia and cavernous sinus syndrome. However, in our patient, the significant direct invasion of both orbits obscured any underlying clinical features related to radiologically apparent left cavernous sinus involvement. Bilateral blindness was due to direct orbital spread of disease, evident on imaging, and confirmed by a histological diagnosis of optic nerve necrosis due to invasion, rather than ischemia due to ophthalmic artery thrombosis. Blindness due to bilateral ophthalmic artery occlusion by mucormycosis was reported by Song and Shin in a single case report [14]. In this report, the patient died 4 days after the admission.

In general, the poor prognosis indicators in ROCM include delayed treatment, intracranial extension, palatal, and orbital involvement [15]. Moreover, vascular extension of the disease would reduce the chance of effective therapy and might worsen the prognosis. With our patient, despite the aggressive management, only a transient benefit has been achieved.

## 4. Conclusion

ROCM-associated vasculopathy can be extensive with no related symptoms, which can make the treatment more difficult. On the other hand, arterial occlusion might suggest delayed diagnosis and more widely spread disease that in turn tends to worsen the prognosis.
